# Prior hip arthroscopy does not affect 1-year patient-reported outcomes following total hip arthroplasty: a register-based matched case-control study of 675 patients

**DOI:** 10.1080/17453674.2021.1884795

**Published:** 2021-02-10

**Authors:** Ida Lindman, Jonatan Nåtman, Axel Öhlin, Karin Svensson Malchau, Louise Karlsson, Maziar Mohaddes, Ola Rolfson, Mikael Sansone

**Affiliations:** a Department of Orthopedics, Institute of Clinical Sciences, Sahlgrenska Academy, University of Gothenburg , Gothenburg ;; b Centre of Registers, Västra Götalandsregionen , Gothenburg ;; c Department of Orthopedics, Sahlgrenska University Hospital , Gothenburg , Sweden

## Abstract

Background and purpose — Femoroacetabular impingement syndrome (FAIS) is a common cause of hip pain and may contribute to the development of osteoarthritis. We investigated whether a prior hip arthroscopy affects the patient-reported outcomes (PROMs) of a later total hip arthroplasty (THA).

Patients and methods — Patients undergoing hip arthroscopy between 2011 and 2018 were identified from a hip arthroscopy register and linked to the Swedish Hip Arthroplasty Register (SHAR). A propensity-score matched control group without a prior hip arthroscopy, based on demographic data and preoperative score from the EuroQoL visual analogue scale (EQ VAS) and hip pain score, was identified from SHAR. The group with a hip arthroscopy (treated group) consisted of 135 patients and the matched control group comprised 540 patients. The included PROMs were EQ-5D and EQ VAS of the EuroQoL group, and a questionnaire regarding hip pain and another addressing satisfaction. Rate of reoperation was collected from the SHAR. The follow-up period was 1 year.

Results — The mean interval from arthroscopy to THA was 27 months (SD 19). The EQ-5D was 0.81 and 0.82, and EQ VAS was 78 and 79 in the treated group and the matched control group respectively. There were no differences in hip pain, and reported satisfaction was similar with 87% in the treated group and 86% in the matched control group.

Interpretation — These results offer reassurance that a prior hip arthroscopy for FAIS does not appear to affect the short-term patient-reported outcomes of a future THA and indicate that patients undergoing an intervention are not at risk of inferior results due to their prior hip arthroscopy.

Femoroacetabular impingement syndrome (FAIS) implies abnormal morphology on the femoral or acetabular side of the hip joint and is a common cause of hip pain and dysfunction in the young population (Matar et al. 2019, Zhou et al. 2020). It reportedly increases the risk of developing osteoarthritis (OA), presumably due to damage to the chondrolabral structures (Ganz et al. 2003, Beck et al. 2005).

Arthroscopic treatment of FAIS has been proven successful with 1- and 5-years’ follow-up (Griffin et al. 2018, Ohlin et al. 2020). However, one of the most common reoperations is conversion to a total hip arthroplasty (THA) (Harris et al. 2013). Depending on the follow-up period and severity of chondrolabral damages, 3–50% of patients with a previous hip arthroscopy for FAIS are reported to undergo THA later in life (Harris et al. 2013).

Whether a prior hip arthroscopy affects the result of a subsequent THA (Haughom et al. 2016, Charles et al. 2017, Perets et al. 2017, Hoeltzermann et al. 2019, Vovos et al. 2019) has previously been discussed. However, many of these studies have been underpowered and the results have been incongruent. Most studies suggested no differences in outcomes in THA for patients with a prior hip arthroscopy (Haughom et al. 2016, Charles et al. 2017, Hoeltzermann et al. 2019). Yet inferior patient satisfaction and higher complication rates were reported in some studies (Perets et al. 2017, Vovos et al. 2019).

To optimize the results for patients undergoing THA surgery, it is important to understand factors that could affect the outcomes. The possible effect of hip arthroscopy on future THA should also be considered during patient selection.

We investigated the influence of a prior hip arthroscopy on a subsequent THA with patient-reported outcome measures (PROMs) 1 year after THA.

**Figure:**
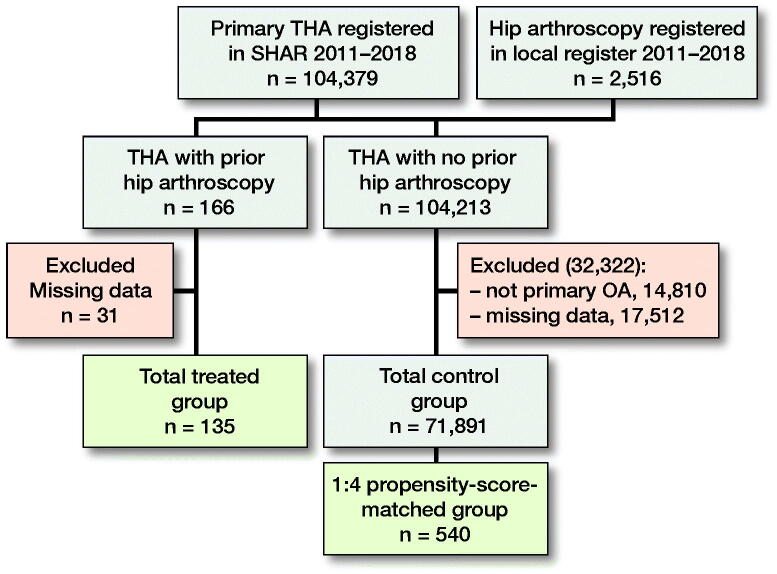
Flow chart of included patients. Excluded diagnoses: tumors, fractures, or trauma. Excluded missing data due to missing preoperatively patient-reported outcomes or demographic data. Abbreviations: SHAR: Swedish Hip Arthroplasty Register, THA: total hip arthroplasty.

## Patients and methods

### Patients

Data were retrieved from a local hip arthroscopy register covering procedures due to FAIS undertaken at 2 hospitals (Sahlgrenska University Hospital, Mölndal and Orthocenter Gothenburg, Sweden) between 2011 and 2018 (Sansone et al. 2014). Based on the unique personal identity number given to all permanent residents in Sweden, this data was linked to the Swedish Hip Arthroplasty Register (SHAR). The SHAR is a national quality register with 98% completeness of registrations for primary THA (Rolfson et al. 2011). There were 135 patients with a subsequent THA in the same hip as the hip arthroscopy identified (treated group). From the SHAR, an overall control group of 71,891 patients with a THA between 2011 and 2018 was created. Only the first operated hip was included in bilaterally operated patients. Patients with a fracture or a tumor as an indication for THA were excluded from the overall control group (Figure). From the overall control group, a 1:4 matched control group with no history of previous hip arthroscopy was further identified.

### Outcome measures

3 different PROMs were used: the EQ-5D-3L health status questionnaire of the EuroQoL-group, a 5-level Likert scale addressing hip pain and a 5-level Likert scale addressing satisfaction with the outcome of THA. These PROMs are part of the routine follow-up program in the SHAR (Rolfson et al. 2011). The EQ-5D index ranges from –0.59 to 1, where 0 is a health state equivalent to death, 1 is equivalent to perfect health, and negative values are considered worse than death (Dolan and Roberts 2002). The EQ-5D further includes a 0 to 100 visual analogue scale (VAS) covering general health (EQ VAS) ranging from 0 to 100. To define the minimal important difference (MID) of the EQ VAS, an improvement of 15 points was used (King 2011). The hip pain Likert scale ranges from 1 (no pain) to 5 (severe pain). The satisfaction item ranges from 1 (very dissatisfied) to 5 (very satisfied). This scale was dichotomized into satisfied (including very satisfied and satisfied) and dissatisfied (very dissatisfied, dissatisfied, and neither satisfied nor dissatisfied). Preoperative and 1-year follow-up PROM data were included in the study. The rate of reoperation was collected from the SHAR.

### Demographic data

Demographic data, such as age at the time of THA surgery, sex, BMI, ASA classification, and type of prosthesis fixation was collected from the SHAR.

### Propensity score matching and control group

For comparisons between potential differences in PROMs at follow-up, a 1:4 propensity-score-matched group was included. The variables included in the propensity matching were age, sex, BMI, ASA classification, type of prothesis fixation, preoperative EQ VAS score, and preoperative hip pain. The treated group consisted of 135 patients with a prior hip arthroscopy, while the matched control group consisted of 540 patients with a THA due to primary OA. To describe and to recognize dissimilarities in the baseline characteristics, demographic data was compared between the treated group and the overall control group of 71,891 patients, as demographic data was included in the propensity match.

Missing data for any of the variables included in the propensity score matching was handled by listwise deletion, meaning that patients with missing data on 1 or more of the variables were excluded from the analysis.

### Statistics

All statistical analyses were performed using R version 3.6.1 (R Centre for Statistical Computing, Vienna, Austria). The outcomes of the 2 groups were compared using the 2-sample t-test for continuous variables and by Pearson’s chi-square test of independence for categorical variables. Results are reported with mean (SD), p-values and 95% confidence intervals (CI). Fisher’s exact test was used for comparing rate of reoperations between the 2 groups. Statistical significance was set at p < 0.05.

Matching was performed using the 1:4 nearest-neighbor matching without replacement. Each treated patient was matched to 4 patients not treated with arthroscopy.

The ability of the propensity-score matching to balance the baseline covariates was assessed using absolute standardized mean differences (SMD). An SMD of < 10% was considered non-significant. For continuous variables, the variances were also compared.

### Ethics, funding, and potential conflicts of interest

The study was approved by the Swedish Ethical Review Authority (number 2019-04682). This study was not financed by any external funding. The authors declare no conflicts of interest.

## Results

In the treated group, 62% were male with a mean age of 51 years, compared with 44% male and a mean age of 68 years in the overall control group (Table 1). The mean time interval to THA from hip arthroscopy was 27 months (SD 19).

**Table 1.** Demographic data presented as numbers (%) or mean values and standard deviations (SD) with standardized mean difference (SMD) between matched control group and treated group

	Overall control n = 71,891	Treated n = 135	Matched control n = 540	SMD (%)
Female sex	40,341 (56)	51 (38)	188 (35)	6.2
ASA classification				
1	17,703 (24)	95 (70)	384 (71)	4.1
2	43,079 (60)	36 (27)	137 (25)	
3–4	11,109 (16)	4 (3)	19 (4)	
Type of prothesis				
Cemented	42,359 (59)	9 (7)	32 (6)	4.0
Hybrid	12,405 (17)	10 (7)	44 (8)	
Uncemented	17,127 (24)	116 (86)	464 (86)	
Mean age (SD)	68 (11)	51 (8)	52 (10)	3.0
Mean BMI (SD)	27 (5)	27 (3)	27 (4)	0

There were no statistically significant differences between the treated group and the matched control group with regard to the EQ-5D or the EQ VAS (Table 2). For the patients undergoing a prior hip arthroscopy, 68% experienced an improvement of 15 points or more on the EQ VAS compared with 65% of the patients in the matched control group.

**Table 2.** Patient-reported outcome for the treated group and the matched control group presented as the mean value, confidence interval (CI), and p-value for Eq-5D, and percentage for hip pain and satisfaction

Variable	Matched control	Treated	Difference 95% CI	p-value
EQ-5D index				
Preoperative	0.35	0.34	–0.05 to 0.07	0.8
1 year postoperative	0.82	0.81	–0.04 to 0.05	0.9
Delta	0.47	0.47	–0.07 to 0.06	0.9
EQ VAS				
Preoperative	51.9	52.4	–5.0 to 3.9	0.8
1 year postoperative	78.8	78.3	–3.2 to 4.2	0.8
Delta	26.9	25.9	–4.1 to 6.1	0.7
Hip pain 1 year postoperatively (%)				
None	55	55		0.7
Very mild	23	21		
Mild	14	15		
Moderate	7	7		
Severe	1	2		
Satisfaction 1 year postoperatively (%)				
Not satisfied	14	13		0.9
Satisfied	86	87		

There was no statistically significant difference between the patients regarding hip pain postoperatively (Table 2). In the treated group and in the matched control group, 94% and 96% of the patients had improved by at least 1 point respectively.

1 year after surgery, 87% of the treated group and 86% of the matched control group were satisfied with surgery (Table 2).

In the treated group, 2 patients were reoperated on during the study period and in the matched control group 19 patients were reoperated on. The reasons for reoperation in the treated group were technical reasons and complications with the implant in the first patient and a deep infection in the second patient, and in the control group the reasons were aseptic loosening (6 patients), deep infection (8 patients), fracture (3 patients), and dislocation (2 patients). The difference regarding the rate of reoperations between the 2 groups was statistically not significant (p = 0.3).

## Discussion

The most important finding in this study was that there were similar patient-reported outcomes between patients undergoing THA after a hip arthroscopy compared with patients without a prior hip arthroscopy. Our findings are similar to those in previous studies, where most report no differences between study groups and control groups at follow-up. A systematic review recently concluded that the short-term outcomes for patients with THA and prior arthroscopy are comparable to those patients undergoing a primary THA; however, many of the included studies were underpowered (Rosinsky et al. 2019). Further, Rosinsky et al. (2020) have reported on the longest follow-up period of 5 years so far. They found no differences in terms of PROMs in a study group comprising 33 patients, yet reported a slightly higher risk of revision for patients who had undergone a prior hip arthroscopy. While Haughom et al. (2016) acknowledged that their study was underpowered for conclusions regarding reoperation, they found the Harris Hip Score (HHS) to be higher preoperatively in the group who had prior hip arthroscopy, while there were no differences in HHS at follow-up. Conversely, Hoeltzermann et al. (2019) found the opposite in terms of the modified HHS (mHHS), which was lower preoperatively in the study group of 33 patients, but neither did they find any differences at follow-up between the group with a prior hip arthroscopy compared with a THA without a prior arthroscopy. Charles et al. (2017) found no differences in postoperative outcomes in a study group comprising 39 patients. Although most studies report no differences between groups, Perets et al. (2017) reported inferior results in terms of a lower HHS, a lower Forgotten Joint Score-12 (FJS-12). and patient satisfaction 2 years after THA surgery in a study group of 35 patients. Further, Vovos et al. (2019), with the largest cohort previously reported, found increased surgical time and increased intraoperative and postoperative complications in a study group of 95 patients with prior hip arthroscopy; however no differences were found regarding revision rates after 2 years’ follow-up. In this study, the number of revisions were few and a prior hip arthroscopy did not increase the risk of reoperation.

We compared demographic data between patients with a history of ipsilateral hip arthroscopy prior to their THA and the overall control group in the SHAR. The group of patients with a prior hip arthroscopy were younger and consisted of more men than the overall control group. The larger proportion of men in the group who had undergone a prior hip arthroscopy is not unexpected, as FAIS is more common in men and a larger proportion of men undergo hip arthroscopy for FAIS (Sansone et al. 2014). One possible theory relating to the younger age of the group with a prior hip arthroscopy is that FAIS is a contributory factor to the development of OA, thereby leading to the need for an earlier THA (Beck et al. 2005). It is still not understood whether the surgical trauma implied by the hip arthroscopy increases or prevents the risk of developing OA and subsequently undergoing a THA. Rhon et al. (2019) found that 22% of the patients undergoing hip arthroscopy had received a clinical diagnosis of OA within 2 years of arthroscopic surgery. In spite of this, it is not known whether these patients would have developed OA regardless of their primary arthroscopic treatment.

Femoroacetabular impingement syndrome is thought to increase the risk of developing osteoarthritis (OA) (Ganz et al. 2003, Beck et al. 2005). However, a study by Wyles et al. (2017) found that the natural history of hips with femoroacetabular impingement morphology was similar to that in hips with normal morphology in terms of the risk of receiving a THA.

It has further been discussed whether arthroscopy for FAIS with concomitant OA could prevent the development of OA and the need for a THA or increase its progression (Ng et al. 2010, Domb et al. 2017). Most studies report improved clinical outcomes for patients undergoing hip arthroscopy for FAIS with concomitant OA (Sansone et al. 2016). However, the indication of hip arthroscopy for OA is debated (Kemp et al. 2015). Nevertheless, patients with severe OA and higher age at the time of hip arthroscopy have been shown to have inferior outcomes and a higher risk of undergoing a THA (Kemp et al. 2015). A previous study found a conversion rate of 68% within 2 years and an increased risk of revision and reoperation in patients undergoing hip arthroscopy, though the indication for the patients in that study was OA (Malahias et al. 2020). The main indication for all patients undergoing arthroscopic surgery in our study was FAIS.

As the indications for arthroscopy evolve, it is important that the indication for surgery is carefully considered. Based on the findings in this study, undergoing hip arthroscopy for the diagnosis of FAIS prior to a THA will not negatively affect the outcome of the THA. In accordance with previous literature, the short-term outcomes after THA are similar for patients with a prior hip arthroscopy.

To our knowledge, this study has the largest study cohort reported, with 135 patients in the treatment group who were compared, after a 1:4 matching, with 540 control patients. The careful matching procedure including both demographic data and PROMs regarding hip pain and general health adds to the strength of the study. Furthermore, the SHAR has a high response rate covering 98% of all THAs performed in Sweden. However, there are limitations to this study. The study does not include intraoperative findings or surgical time. Nor does the study include the specific grade of OA prior to hip arthroscopic surgery; however, the indication for hip arthroscopic surgery was not OA in any patient. The local hip arthroscopy register includes patients undergoing a hip arthroscopy in the western part of Sweden, generating a possible risk of patients in the matched control group having undergone a prior hip arthroscopy in other parts of the country not covered by this register. Further, patients were excluded prior to the propensity-score matching due to missing data. There is always a risk of bias with missing data, but these patients should not be considered as dropouts as they were not fulfilling the requirement for inclusion in this study. Another limitation is that no sample size calculation was performed prior to the study, as all the patients who underwent hip arthroscopy prior to their THA included in the SHAR were included in this study. Although the cohort is larger than that in previous studies evaluating hip arthroscopy prior to THA, there is still a risk of type 2 error. This study reports outcomes 1 year after undergoing a THA and it would be interesting to follow the cohort for a longer period.

In conclusion, prior hip arthroscopy for FAIS does not appear to affect the patient-reported outcomes of a future THA. In the decision to undergo hip arthroscopy, these results offer reassurance that such an intervention is not likely to influence patient-reported outcomes after an eventual future THA and indicate that patients are not at risk of inferior results due to their prior hip arthroscopy.
